# Association of bariatric surgery with risk of acute care use for hypertension-related disease in obese adults: population-based self-controlled case series study

**DOI:** 10.1186/s12916-017-0914-5

**Published:** 2017-08-23

**Authors:** Yuichi J. Shimada, Yusuke Tsugawa, Hiroyasu Iso, David F. M. Brown, Kohei Hasegawa

**Affiliations:** 1 Cardiology Division, Department of Medicine, Massachusetts General Hospital, Harvard Medical School, 55 Fruit Street, Gray/Bigelow 800, Boston, MA 02114 USA; 2 Department of Health Policy and Management, Harvard T.H. Chan School of Public Health, 677 Huntington Ave, Boston, MA 02115 USA; 3 Public Health, Department of Social Medicine, Osaka University Graduate School of Medicine, 1-1 Yamadaoka, Suita, Osaka 565-0871 Japan; 4 Department of Emergency Medicine, Massachusetts General Hospital, Harvard Medical School, 55 Fruit Street, Boston, MA 02114 USA

**Keywords:** Bariatric surgery, Emergency department visit, Hospitalization, Hypertension, Obesity, Self-controlled case series study

## Abstract

**Background:**

Hypertension carries a large societal burden. Obesity is known as a risk factor for hypertension. However, little is known as to whether weight loss interventions reduce the risk of hypertension-related adverse events, such as acute care use (emergency department [ED] visit and/or unplanned hospitalization). We used bariatric surgery as an instrument for investigating the effect of large weight reduction on the risk of acute care use for hypertension-related disease in obese adults with hypertension.

**Methods:**

We performed a self-controlled case series study of obese patients with hypertension who underwent bariatric surgery using population-based ED and inpatient databases that recorded every bariatric surgery, ED visit, and hospitalization in three states (California, Florida, and Nebraska) from 2005 to 2011. The primary outcome was acute care use for hypertension-related disease. We used conditional logistic regression to compare each patient's risk of the outcome event during sequential 12-month periods, using pre-surgery months 13–24 as the reference period.

**Results:**

We identified 980 obese patients with hypertension who underwent bariatric surgery. The median age was 48 years (interquartile range, 40–56 years), 74% were female, and 55% were non-Hispanic white. During the reference period, 17.8% (95% confidence interval [CI], 15.4–20.2%) had a primary outcome event. The risk remained unchanged in the subsequent 12-month pre-surgery period (18.2% [95% CI, 15.7–20.6%]; adjusted odds ratio [aOR] 1.02 [95% CI, 0.83–1.27]; *P* = 0.83). In the first 12-month period after bariatric surgery, the risk significantly decreased (10.5% [8.6–12.4%]; aOR 0.58 [95% CI, 0.45–0.74]; *P* < 0.0001). Similarly, the risk remained significantly reduced in the 13–24 months after bariatric surgery (12.9% [95% CI, 10.8–15.0%]; aOR 0.71 [95% CI, 0.57–0.90]; *P* = 0.005). By contrast, there was no significant reduction in the risk among obese patients who underwent non-bariatric surgery (i.e., cholecystectomy, hysterectomy, spinal fusion, or mastectomy).

**Conclusions:**

In this population-based study of obese adults with hypertension, we found that the risk of acute care use for hypertension-related disease decreased by 40% after bariatric surgery. The data provide the best evidence on the effectiveness of substantial weight loss on hypertension-related morbidities, underscoring the importance of discussing options for weight reduction when treating obese patients with hypertension.

**Electronic supplementary material:**

The online version of this article (doi:10.1186/s12916-017-0914-5) contains supplementary material, which is available to authorized users.

## Background

Hypertension (HTN) is the leading public health problem, affecting more than one billion adults worldwide [1]. In the USA, the prevalence of HTN in adults is approximately 33% (80.0 million Americans) with estimated direct and indirect costs of $49 billion in 2012 [2]. Among these patients, blood pressure is uncontrolled in approximately half of them, particularly in obese individuals [3, 4]. HTN accounts for a substantial healthcare utilization, e.g., one million emergency department (ED) visits and 500,000 hospitalizations annually [2]. In parallel, the USA has also experienced an obesity epidemic — 35% of men and 40% of women are obese [5]. Significant weight reduction is known to decrease blood pressure and sometimes results in remission of HTN among obese adults [6–10]. Among various weight management strategies, bariatric surgery is the most effective method to achieve substantial and sustained weight loss [11]. However, little is known about the impact of significant weight reduction with bariatric surgery on acute care use (ED visits and/or unplanned hospitalizations) for HTN-related disease [12].

In this context, we aimed to determine whether bariatric surgery, as an instrument to achieve large weight reduction, reduces the risk of acute care use for HTN-related disease among obese patients with HTN. To do this, we used large longitudinal datasets from three diverse states. A better understanding of the role of bariatric surgery in the prevention of HTN-associated morbidities would provide further insight into therapeutic strategies for obese patients with HTN.

## Methods

### Study design and setting

We performed a self-controlled case series study using the Healthcare Cost and Utilization Project (HCUP) State Emergency Department Databases (SEDD) and State Inpatient Databases (SID) [13, 14]. The study design was selected because each patient serves as his/her own control; therefore, a separate control group is not necessary [15]. This study performed intra-person comparisons in the patients who experienced both the exposure (bariatric surgery) and the outcome (acute care use for HTN-related disease). Confounding by unmeasured variables was minimized, as all time-invariant covariates (e.g., patient characteristics and genetics) were implicitly controlled [15].

We analyzed the data from HCUP SEDD and SID in three states (California, Florida, and Nebraska) from 2005 to 2011. The HCUP is the largest longitudinal hospital care database in the USA and provides all-payer, encounter-level information [13, 14]. The SEDD records all ED visits including treat-and-release encounters and transfers from short-term, acute-care, and nonfederal hospitals in participating states [13]. The HCUP SID captures all inpatient discharges from short-term, acute-care, nonfederal, general, and other specialty hospitals [14]. Integration of HCUP SEDD and SID enables us to identify all ED visits regardless of disposition and all hospitalizations regardless of the source of hospitalization in the three states [13, 14]. We chose these three states because they are geographically diverse and have unique patient identifiers that enabled us to perform longitudinal patient follow-up across the study years within the states. Details of the study design, databases, and statistical methods have been published elsewhere [13, 14, 16–19]. The institutional review board of Massachusetts General Hospital approved this study.

### Study population

The following steps were undertaken to identify all obese adults (aged ≥18 years) who underwent bariatric surgery and had an acute care use for HTN-related disease in the three states. First, we identified adults with a diagnosis code for obesity who had at least one hospitalization for bariatric surgery. The International Classification of Diseases, Ninth Revision, Clinical Modification (ICD-9-CM) diagnosis codes for obesity were 278.0–278.2, V77.8, V85.3x, and V85.4 [16–20]. The Current Procedural Terminology codes for bariatric surgery were 43.89, 44.31, 44.38, 44.39, 44.50, 44.68, 44.69, 44.93, 44.95, 44.99, 45.51, and 45.90 [16–20]. We excluded patients who had diagnostic codes for gastrointestinal cancer (ICD-9-CM codes 150.0-159.9) [16–19]. To allow for data collection during the 2-year pre-surgery and post-surgery periods, we included patients who underwent bariatric surgery between 1 January 2007 and 31 December 2009. Second, we further identified patients with HTN, which was defined in the present study as having at least one acute care use for HTN-related disease during the study years, i.e., between 1 January 2005 and 31 December 2011. The ICD-9-CM diagnosis codes for HTN-related disease were 401 (essential hypertension), 402 (hypertensive heart disease), 403 (hypertensive chronic kidney disease), 404 (hypertensive heart and chronic kidney disease), 405 (secondary hypertension), and 437.2 (hypertensive encephalopathy), as the primary diagnosis [21, 22]. The exclusion criteria were residents outside the three states and patients who died during the hospitalization for bariatric surgery, had an in-hospital death during the 2-year post-surgery period, or had multiple bariatric surgeries during the study period. We also excluded planned hospitalizations to examine acute care needs in patients with HTN.

### Measurements

We used the baseline characteristics recorded during the index hospitalization for bariatric surgery. We retrieved demographics data including age, sex, and race/ethnicity (non-Hispanic white, non-Hispanic black, Hispanic, and “other”), primary insurance type (Medicare, Medicaid, private sources, and “other”), quartiles for estimated median household income of residents in the patient’s ZIP code, ICD-9-CM diagnosis, procedures, disposition, season of surgery, and state.

### Statistical analysis

The primary endpoint was a composite of ED visit or unplanned hospitalization with a primary diagnosis related to HTN during a 4-year period (i.e., 2 years before and 2 years after bariatric surgery). We computed adjusted odds ratios (aORs) with a conditional logistic regression model using pre-surgery months 13–24 as the reference period for 1–12 months before surgery, 0–12 months after surgery, and 13–24 months after surgery. Each patient was matched to his/her own reference period.

We tested the robustness of our inferences by performing several sensitivity analyses. First, we repeated the analysis stratified by age group (18–44, 45–54, and ≥55 years) and sex. Second, we modeled the primary endpoint as a count variable as opposed to a binary outcome with a negative binomial regression model. Third, we repeated the primary model in a subgroup of patients who had at least one acute care use for any reason during post-surgery 25–36 months. This sensitivity analysis addressed the possibility of loss to follow-up (e.g., out-of-hospital deaths, moving out of the study states). This subgroup selection method ensured that these patients were both alive and living within the study states at least until 2 years after surgery and would have been recorded in the databases if they had the primary endpoint during the study period. Fourth, we conducted a sensitivity analysis using a more restrictive definition of the outcome event, i.e., only including the ICD-9-CM diagnosis codes 401 and 437.2. Fifth, we performed the self-controlled case series analysis for four other types of elective surgery: cholecystectomy (Current Procedural Terminology codes 51.21–51.24 and 51.41–51.59), hysterectomy (Current Procedural Terminology codes 68.31–68.79 and 68.9), spinal fusion (Current Procedural Terminology codes 81.00–81.66), and mastectomy (Current Procedural Terminology codes 85.41–85.48) [16, 17]. We performed this analysis to address the possibility that reductions in the risk of acute care use for HTN-related disease might be observed with any elective surgery in general (e.g., intensified blood pressure control during peri-surgical period). We selected these non-bariatric surgeries because they have a large sample size, similar characteristics (i.e., common elective surgery), and no biological plausibility to affect weight or the risk of acute care use for HTN-related disease. Lastly, to delineate the differential effects of individual types of bariatric surgery on the risk of acute care use for HTN-related disease, we performed separate self-controlled case series analyses for the two most common types of bariatric surgery: gastric bypass (Current Procedural Terminology codes 44.31, 44.38, and 44.39) and gastric banding (Current Procedural Terminology codes 44.68 and 44.95) [23]. All analyses were performed at a two-sided significance level of 0.05, and all confidence intervals (CIs) were reported as two-sided values with a confidence level of 95%. Statistical analyses were performed with SAS version 9.4 (SAS Institute, Cary, NC, USA).

## Results

We identified a total of 1022 obese adults who underwent bariatric surgery between 1 January 2007 and 31 December 2009 and also had at least one ED visit or hospitalization with a primary diagnosis related to HTN between 1 January 2005 and 31 December 2011. From this population, we excluded 18 patients who had an in-hospital death within 2 years after bariatric surgery and 26 patients who underwent multiple bariatric surgeries (two patients had both). We included the remaining 980 patients in the primary analysis. Table 1 describes the baseline characteristics at the time of bariatric surgery. The median age was 48 years (interquartile range, 40–56 years), 74% were female, and 55% were non-Hispanic white.

**Table 1:** Baseline characteristics of patients with hypertension who underwent bariatric surgery

Characteristics	Number (*n*) = 980
Age (years), median (IQR)	48 (40–56)
Female sex	718 (73.6)
Race/ethnicity^a^	
Non-Hispanic white	512 (54.8)
Non-Hispanic black	254 (27.2)
Hispanic	142 (15.2)
Other	26 (2.8)
Primary insurance	
Medicare	240 (24.5)
Medicaid	108 (11.0)
Private	560 (57.2)
Other	71 (7.3)
Quartiles for median household income of patient's ZIP code	
1 (lowest)	301 (31.1)
2	265 (27.4)
3	235 (24.3)
4 (highest)	167 (17.3)
Season of bariatric surgery	
January–March	204 (20.8)
April–June	234 (23.9)
July–September	281 (28.7)
October–December	261 (26.6)
State	
California	600 (61.2)
Florida	368 (37.6)
Nebraska	12 (1.2)

Data were expressed as numbers (percentages), unless otherwise indicated. *IQR* interquartile range

^a^Analyzed for 934 (95.3%) patients with race/ethnicity data. Race/ethnicity data were not available in Nebraska

**Fig. 1:**
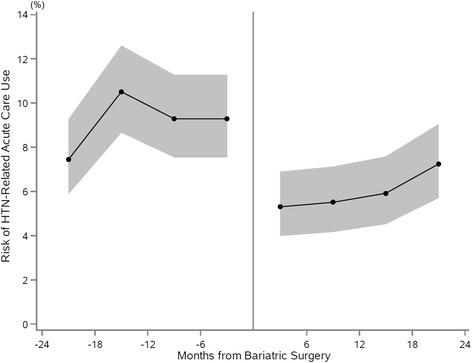
Risk of acute care use for hypertension-related disease before and after bariatric surgery in a 6-month interval. Shown is the proportion of patients with an acute care use (ED visit and/or unplanned hospitalization) related to HTN with the 95% CIs for the 2 years before and after bariatric surgery in 6-month intervals. The periods were centered on the date of bariatric surgery of each patient. *CI* confidence interval, *ED* emergency department, *HTN* hypertension

Table 2 summarizes the risk of acute care use for HTN-related disease in the pre- and post-bariatric surgery periods. During the reference period (i.e., 13–24 months prior to bariatric surgery), we observed at least one acute care use for HTN-related disease in 17.8% (95% CI 15.4–20.2%) of the study population. The risk did not change in the following 12-month pre-surgery period (18.2%, 95% CI 15.7–20.6%), corresponding to an aOR of 1.02 (95% CI 0.83–1.27; *P* = 0.83). By contrast, we observed a significant decline in the risk after bariatric surgery. Within 12 months after bariatric surgery, 10.5% (95% CI 8.6–12.4%) experienced an acute care use for HTN-related disease (aOR 0.58, 95% CI 0.45–0.74; *P* < 0.0001). The risk remained significantly reduced during the subsequent period of 13–24 months post-surgery (12.9%, 95% CI 10.8–15.0%), corresponding to an aOR of 0.71 (95% CI 0.57–0.90; *P* = 0.005; Fig. 1).

**Table 2:** Number of patients and risk of acute care use for hypertension-related disease

Time interval and outcome	Number of patients	Risk, % (95% CI)	aOR (95% CI)^a^	*P* value
	(*n* = 980)			
13–24 months before bariatric surgery				
ED visit or hospitalization^b^	174	17.8 (15.4–20.2)	Reference	–
ED visit^c^	130	13.3 (11.1–15.4)	Reference	–
Hospitalization^d^	44	4.5 (3.2–5.8)	Reference	–
1–12 months before bariatric surgery				
ED visit or hospitalization^b^	178	18.2 (15.7–20.6)	1.02 (0.83–1.27)	0.83
ED visit^c^	130	13.3 (11.1–15.4)	1.00 (0.78–1.28)	0.99
Hospitalization^d^	50	5.1 (3.7–6.5)	1.14 (0.76–1.73)	0.53
0–12 months after bariatric surgery				
ED visit or hospitalization^b^	103	10.5 (8.6–12.4)	0.58 (0.45–0.74)	<0.0001
ED visit^c^	91	9.3 (7.5–11.1)	0.69 (0.53–0.91)	0.008
Hospitalization^d^	14	1.4 (0.7–2.2)	0.31 (0.17–0.57)	<0.0001
13–24 months after bariatric surgery				
ED visit or hospitalization^b^	126	12.9 (10.8–15.0)	0.71 (0.57–0.90)	0.005
ED visit^c^	100	10.2 (8.3–12.1)	0.76 (0.58–0.99)	0.04
Hospitalization^d^	30	3.1 (2.0–4.1)	0.68 (0.42–1.08)	0.10

*CI* confidence interval, *aOR* adjusted odds ratio, *ED* emergency department

^a^Adjusted odds ratios are for each 12-month period versus the reference period (i.e., 13–24 months before the index bariatric surgery), as calculated with conditional logistic regression

^b^At least one acute care use (ED visit or unplanned hospitalization) for HTN-related disease

^c^At least one ED visit for HTN-related disease, not resulting in hospitalization

^d^At least one unplanned hospitalization for HTN-related disease

In the stratification analysis by age group with a limited statistical power, a similar risk reduction was observed in the 18–44 and 45–54 year age groups (Additional file 1). The sensitivity analysis stratified by sex showed that women had a similar reduction in the risk over the 2-year post-surgery period, while men had a significant reduction only in the first 12 months after bariatric surgery (Additional file 2). The sensitivity analysis modeling the outcome as a count variable replicated the findings of the main analysis (Additional file 3). Likewise, the subgroup analysis of patients who had any acute care use during 25–36 months after bariatric surgery (*n* = 325) showed, even with a limited statistical power, a similar pattern with a larger effect size (Table 3). The sensitivity analysis using a more restrictive definition of HTN-related disease demonstrated consistent results (Additional file 4). By contrast, in the separate self-controlled case series analyses with obese patients with HTN who underwent cholecystectomy (*n* = 378), hysterectomy (*n* = 112), spinal fusion (*n* = 61), or mastectomy (*n* = 30), the risk of the primary outcome did not decrease after non-bariatric surgery (Table 4). The sensitivity analysis according to the type of bariatric surgery showed that the point estimate of the odds ratio was lower after gastric bypass compared to gastric banding (Additional file 5).

**Table 3:** Number of patients and risk of acute care use for hypertension-related disease, limiting to patients with any healthcare utilization during 25–36 months after bariatric surgery

	Number of patients	Risk, % (95% CI)^a^	aOR (95% CI)^b^	*P* value
Time interval	(*n* = 325)			
13–24 months before bariatric surgery	59	18.2 (13.9–22.4)	Reference	–
1–12 months before bariatric surgery	52	16.0 (12.0–20.0)	0.87 (0.59–1.29)	0.49
0–12 months after bariatric surgery	29	8.9 (5.8–12.0)	0.47 (0.30–0.74)	0.001
13–24 months after bariatric surgery	41	12.6 (9.0–16.2)	0.68 (0.45–1.02)	0.06

*CI* confidence interval, *aOR* adjusted odds ratio

^a^At least one acute care use (ED visit or unplanned hospitalization) for HTN-related disease

^b^Adjusted odds ratios are for each 12-month period versus the reference period (i.e., 13–24 months before the index bariatric surgery), as calculated with conditional logistic regression

**Table 4:** Number of patients and risk of acute care use for hypertension-related disease among obese patients with hypertension who underwent non-bariatric surgery

Time interval and surgery	Number of patients	Risk, % (95% CI)^a^	aOR (95% CI)^b^	*P* value
Cholecystectomy	(*n* = 378)			
13–24 months before surgery	64	16.9 (13.1–20.7)	Reference	–
1–12 months before surgery	129	34.1 (29.3–38.9)	2.13 (1.56–2.91)	<0.0001
0–12 months after surgery	135	35.7 (30.9–40.6)	2.24 (1.65–3.06)	<0.0001
13–24 months after surgery	67	17.7 (13.9–21.6)	1.05 (0.74–1.49)	0.79
Hysterectomy	(*n* = 112)			
13–24 months before surgery	20	17.9 (10.7–25.1)	Reference	–
1–12 months before surgery	42	37.5 (28.4–46.6)	2.26 (1.29–3.95)	0.004
0–12 months after surgery	34	30.4 (21.7–39.0)	1.78 (1.002–3.18)	0.049
13–24 months after surgery	21	18.8 (11.4–26.1)	1.05 (0.56–1.99)	0.87
Spinal fusion	(*n* = 61)			
13–24 months before surgery	11	18.0 (8.1–28.0)	Reference	–
1–12 months before surgery	20	32.8 (20.7–44.9)	1.92 (0.89–4.14)	0.10
0–12 months after surgery	17	27.9 (16.3–39.4)	1.60 (0.73–3.53)	0.24
13–24 months after surgery	11	18.0 (8.1–28.0)	1.00 (0.42–2.37)	0.99
Mastectomy	(*n* = 30)			
13–24 months before surgery	5	16.7 (25.1–30.8)	Reference	–
1–12 months before surgery	11	36.7 (18.4–55.0)	2.34 (0.78–6.98)	0.13
0–12 months after surgery	9	30.0 (12.6–47.4)	1.87 (0.61–5.78)	0.28
13–24 months after surgery	5	16.7 (25.1–30.8)	1.00 (0.28–3.55)	0.99

*CI* confidence interval, *aOR* adjusted odds ratio

^a^At least one acute care use (ED visit or unplanned hospitalization) for HTN-related disease

^b^Adjusted odds ratios are for each 12-month period versus the reference period (i.e., 13–24 months before surgery), as calculated with conditional logistic regression

## Discussion

### Principal findings

By using population-based data of patients with HTN who underwent bariatric surgery in the three diverse states, we found that the risk of acute care use for HTN-related disease decreased by 40% after bariatric surgery. The observed large decline in the risk remained significant for at least 2 years after surgery. In contrast, other non-bariatric surgeries were not associated with a reduced risk of acute care use for HTN-related disease, addressing the possibility that the observed decrease in the risk might be attributable to intensified blood pressure control during the peri-surgical period.

### Results in context

Several studies have reported that large weight reduction by surgical interventions lowers blood pressure and sometimes leads to remission of HTN [6–10]. However, the inferences from these studies on HTN-related healthcare utilization are potentially limited by the lack of assessment of acute care utilization (e.g., ED visit or hospitalization) related to HTN. By contrast, in the present study, both the population and the outcome are unique, because all patients had at least one ED visit or hospitalization for HTN-related disease, and the risk of such healthcare utilization was assessed. This study adds to the body of knowledge by demonstrating the effectiveness of substantial weight reduction (bariatric surgery as an instrument) on the risk of hospital-based acute care use for HTN-related disease among obese patients with HTN.

Physiological studies in humans have indicated that a substantial weight loss can reverse some of the links between obesity and HTN-related morbidities. For instance, weight loss intervention has been reported to improve blood pressure control with a dose–response relationship [24, 25]. Bariatric surgery has been known to favorably affect endothelial function, systemic inflammation, and oxidative stress [24–28]. Moreover, the present study demonstrated that bariatric surgery was associated with a substantially and persistently lower risk of acute care use for HTN-related disease. Additionally, gastric bypass surgery, which is known to result in a larger weight loss (~60% excess weight loss) than gastric banding (~35% excess weight loss) [29], may achieve a larger risk reduction compared to gastric banding. Our data, along with prior evidence, collectively indicate that substantial weight loss may reverse the link between obesity and HTN-related morbidities.

### Advantages of the study design

The self-controlled case series design augments the internal validity, because it eliminates inter-personal variations and enables a precise assessment of impact of the exposure (i.e., bariatric surgery) [15]. In addition, in contrast to the traditional case–control or other cohort study designs, confounding by time-invariant variables (both measured and unmeasured) is addressed, as each subject functions as a control for her/himself [15]. Because of these advantages, the self-controlled case series study design has been successfully used to demonstrate the associations between weight reduction and morbidity in other disease conditions (e.g., congestive heart failure, asthma, and stable angina pectoris) [16–18]. The present study meets the requirements of the self-controlled case series design, because the exposure is transient and discrete and the outcome is an acute event [15].

With regard to the external validity, it has been reported that subjects participating in randomized controlled trials (RCTs) may be highly selected or behave differently compared to the general populations in the real-world setting [30, 31]. For instance, most of the previously published RCTs on bariatric surgery enrolled <10% of the screened patients [32, 33]. By contrast, the patients analyzed in the present study have diverse racial/ethnic, socioeconomic, and geographic characteristics with the use of large population-based databases that capture all ED visits and hospitalizations in the studied states. This diversity in the study population strengthens the external validity of our inferences.

### Possible differential effects according to age group and sex

In the sensitivity analysis stratifying patients by age group, we observed similar risk reduction of acute care use for HTN-related disease after bariatric surgery in the younger populations. This finding is mirrored by a prior retrospective cohort study in patients who underwent bariatric surgery. That study reported that improvement in HTN control was observed in a significantly greater proportion of patients <60 years of age compared to patients ≥60 years of age [34]. In addition, although we observed that the direction and effect size of bariatric surgery on the risk of acute care use for HTN-related disease during the first year after bariatric surgery was similar across female and male patients, the effect persisted longer in female patients. The potential reasons for this discrepancy are likely multifactorial, such as differences in health behaviors [35, 36], access to healthcare [37–40], pre-existent cardiovascular comorbidities [41–43], or any combination of these factors [44]. For example, older and male patients might have had more established arterial atherosclerosis which would be less reversible with weight loss [45, 46]. Our observations suggest that weight reduction achieved by bariatric surgery may not be sufficient to prevent HTN-associated morbidities among older or male patients with HTN. The results of these stratified analyses underscore the importance of identifying underlying mechanisms that account for the differential effects of bariatric surgery and developing targeted strategies for the patient populations in whom bariatric surgery may be less effective.

### Accuracy of case, exposure, and outcome identification

Although the HCUP databases have been widely used in the literature, and the quality has been extensively tested [16–19, 47], misclassification is possible as with any studies using administrative data. With regard to obesity, the specificity of the ICD-9-CM codes for obesity was reported to be 99.4% [48]. While the HCUP databases did not include data on body mass index, patients without obesity have therefore been excluded from our study [48]. Regarding the exposure, the method of identifying patients who underwent bariatric surgery has been used in several studies [16–20]. Moreover, it is well documented in the literature that bariatric surgery leads to substantial weight loss [49]. For example, prompt weight loss was observed within a few months after bariatric surgery and persisted for 12 to 18 months, with a mean weight loss of 35% [49]. These data support the idea that bariatric surgery can be used as an effective method to achieve significant weight reduction. With respect to the outcome, the set of ICD-9-CM codes has been used in previously published studies, and this combination has been shown to have a specificity of 95% and a positive predictive value of 97% for identifying HTN-related ED visits [21, 22]. Thus, our outcome identification strategy has effectively excluded acute care use for conditions other than HTN-related disease.

### Potential limitations

Our study has several potential limitations. First, the probability of exposure may have been affected by a previous outcome event — for instance, patients who had an acute care use for HTN-related disease during the pre-operative period would not have undergone bariatric surgery while blood pressure was poorly controlled. However, this would have decreased the risk in the pre-surgery period and hence biased the inferences toward the null [15]. Second, we determined the risk of acute care use for HTN-related disease by ED visits or unplanned hospitalizations [12]. Therefore, one might argue that there might have been a compensatory increase in less acute forms of healthcare utilization for HTN (e.g., walk-in clinic, urgent care, and other ambulatory care visits). However, we also observed a significant reduction in the risk of hospitalizations, arguing against this possibility. Third, patients might have been lost to follow-up after bariatric surgery, thereby downwardly biasing our estimates. However, the sensitivity analysis limiting to patients who were confirmed to be alive and living within the study states for at least 2 years after surgery showed consistent findings. Fourth, the HCUP database did not include several potentially useful parameters such as information on diet, exercise, medication use, and patient education. Therefore, one might surmise that the observed reduction in the risk may be a result of intensified HTN management during the peri-surgical period and may not be unique to bariatric surgery. However, it would be difficult to postulate that the risk reduction was fully attributable to intensified antihypertensive treatment for the following reasons: (1) a number of prior studies have reported that the majority of patients were able to either discontinue or decrease the number of antihypertensive medications after bariatric surgery [2–6], (2) no reduction in the risk was observed among obese adults with HTN who underwent elective non-bariatric surgery, and (3) there was no signal of risk reduction immediately prior to bariatric surgery when the medical regimen for HTN was likely optimized. It is possible that medication compliance might have improved after bariatric surgery. Finally, the inferences from our study may not be generalizable to other countries with optimal access to and resources in the primary care setting.

## Conclusions

This self-controlled case series study using large population-based datasets from three US states demonstrated that bariatric surgery is associated with a significant reduction in the risk of acute care use for HTN-related disease among obese adults with HTN. However, a large proportion of obese adults with HTN would choose not to undergo bariatric surgery for various reasons, such as an absence of indications, lack of insurance coverage, and peri-surgical risk. Our data also underscore the importance of developing safe and effective noninvasive weight loss strategies for obese patients with HTN to relieve the large societal burden of acute care use for HTN-related disease. Such effort should progress in concert with public health interventions to primarily prevent obesity and HTN to begin with.

## Additional files

Additional file 1: Number of patients and risk of acute care use for hypertension-related disease, stratified by age group. (DOCX 27 kb)

Additional file 2: Number of patients and risk of acute care use for hypertension-related disease, stratified by sex. (DOCX 27 kb)

Additional file 3: Rate ratios for acute care use for hypertension-related disease, using negative binomial regression model. (DOCX 26 kb)

Additional file 4: Number of patients and risk of acute care use for hypertension-related disease, with more restrictive definition. (DOCX 27 kb)

Additional file 5: Number of patients and risk of acute care use for hypertension-related disease with gastric bypass and gastric banding. (DOCX 27 kb)

## Abbreviations

aOR: Adjusted odds ratio
CI: Confidence interval
ED: Emergency department
HCUP: Healthcare Cost and Utilization Project
HTN: Hypertension
ICD-9-CM: International Classification of Diseases, Ninth Revision, Clinical Modification
RCT: Randomized controlled trial
SEDD: State Emergency Department Databases
SID: State Inpatient Databases
