# Supplementary material for: Association of bariatric surgery with risk of acute care use for hypertension-related disease in obese adults: population-based self-controlled case series study
Source: BMC Med. 2017 Aug 23;15:161. doi: 10.1186/s12916-017-0914-5 (PMC5568280; doi:10.1186/s12916-017-0914-5)
Supplement: Supplementary file 1 — Number of patients and risk of acute care use for hypertension-related disease, stratified by age group. (DOCX 27 kb) [file 12916_2017_914_MOESM1_ESM.docx]

**Additional file 1. Number of Patients and Risk of Acute Care Use for Hypertension-related Disease, Stratified by Age Group**

| **Time interval and age group** | **Number of patients** | **Risk, % (95% CI)*** | **aOR (95% CI)**† | **P value** |
| --- | --- | --- | --- | --- |
| **13-24 months before bariatric surgery** |  |  |  |  |
| 18-44 years (n=369) | 77 | 20.9 (16.7-25.0) | reference | - |
| 45-54 years (n=309) | 52 | 16.8 (12.6-21.0) | reference | - |
| 55+ years (n=298) | 45 | 15.1 (11.0-19.2) | reference | - |
| **1-12 months before bariatric surgery** |  |  |  |  |
| 18-44 years | 77 | 20.9 (16.7-25.0) | 1.00 (0.72-1.39) | 0.99 |
| 45-54 years | 51 | 16.5 (12.3-20.7) | 0.98 (0.66-1.45) | 0.92 |
| 55+ years | 49 | 16.4 (12.2-20.7) | 1.09 (0.72-1.65) | 0.67 |
|  |  |  |  |  |
| **0-12 months after bariatric surgery** |  |  |  |  |
| 18-44 years | 37 | 10.0 (6.9-13.1) | 0.46 (0.31-0.69) | <0.0001 |
| 45-54 years | 31 | 10.0 (6.7-13.4) | 0.59 (0.38-0.92) | 0.02 |
| 55+ years | 35 | 11.7 (8.1-15.4) | 0.77 (0.49-1.21) | 0.25 |
| **13-24 months after bariatric surgery** |  |  |  |  |
| 18-44 years | 45 | 12.2 (8.8-15.5) | 0.57 (0.39-0.83) | 0.003 |
| 45-54 years | 34 | 11.0 (7.5-14.5) | 0.65 (0.41-1.003) | 0.052 |
| 55+ years | 47 | 15.8 (11.6-19.9) | 1.05 (0.69-1.59) | 0.83 |

CI, confidence interval; aOR, adjusted odds ratio

* At least one acute care use (ED visit or unplanned hospitalization) for HTN-related disease.

†Adjusted odds ratios are for each 12-month period versus the reference period (i.e., 13-24 months before the index bariatric surgery), as calculated with conditional logistic regression.
