# Supplementary material for: Association of bariatric surgery with risk of acute care use for hypertension-related disease in obese adults: population-based self-controlled case series study
Source: BMC Med. 2017 Aug 23;15:161. doi: 10.1186/s12916-017-0914-5 (PMC5568280; doi:10.1186/s12916-017-0914-5)
Supplement: Supplementary file 2 — Number of patients and risk of acute care use for hypertension-related disease, stratified by sex. (DOCX 27 kb) [file 12916_2017_914_MOESM2_ESM.docx]

**Additional file 2. Number of Patients and Risk of Acute Care Use for Hypertension-related Disease, Stratified by Sex**

| **Time interval and sex** | **Number of patients** | **Risk, % (95% CI)*** | **aOR (95% CI)**† | **P value** |
| --- | --- | --- | --- | --- |
| **Female** | (n=718) |  |  |  |
| 13-24 months before surgery | 134 | 18.7 (15.8-21.5) | reference | - |
| 1-12 months before surgery | 137 | 19.1 (16.2-22.0) | 1.02 (0.80-1.31) | 0.85 |
| 0-12 months after surgery | 80 | 11.1 (8.8-13.4) | 0.58 (0.44-0.77) | <0.0001 |
| 13-24 months after surgery | 86 | 12.0 (9.6-14.4) | 0.63 (0.48-0.83) | 0.001 |
|  |  |  |  |  |
| **Male** | (n=257) |  |  |  |
| 13-24 months before surgery | 40 | 15.6 (11.1-20.0) | reference | - |
| 1-12 months before surgery | 40 | 15.6 (11.1-20.0) | 1.00 (0.64-1.56) | 0.99 |
| 0-12 months after surgery | 23 | 8.9 (5.4-12.5) | 0.57 (0.34-0.95) | 0.03 |
| 13-24 months after surgery | 40 | 15.6 (11.1-20.0) | 1.00 (0.64-1.56) | 0.99 |

CI, confidence interval; aOR, adjusted odds ratio

*At least one acute care use (ED visit or unplanned hospitalization) for HTN-related disease.

†Adjusted odds ratios are for each 12-month period versus the reference period (i.e., 13-24 months before the index bariatric surgery), as calculated with conditional logistic regression.
