# Supplementary material for: Association of bariatric surgery with risk of acute care use for hypertension-related disease in obese adults: population-based self-controlled case series study
Source: BMC Med. 2017 Aug 23;15:161. doi: 10.1186/s12916-017-0914-5 (PMC5568280; doi:10.1186/s12916-017-0914-5)
Supplement: Supplementary file 3 — Rate ratios for acute care use for hypertension-related disease, using negative binomial regression model. (DOCX 26 kb) [file 12916_2017_914_MOESM3_ESM.docx]

**Additional file 3. Rate Ratios for Acute Care Use for Hypertension-related Disease, Using Negative Binomial Regression Model**

| **Time interval** | **Rate Ratio, (95% CI)*** | **P value** |
| --- | --- | --- |
| **ED visit or hospitalization**† |  |  |
| 13-24 months before bariatric surgery | reference | - |
| 1-12 months before bariatric surgery | 1.01 (0.82-1.24) | 0.92 |
| 0-12 months after bariatric surgery | 0.58 (0.45-0.74) | <0.0001 |
| 13-24 months after bariatric surgery | 0.73 (0.57-0.93) | 0.009 |

CI, confidence interval; ED, emergency department

*Rate ratios are for each 12-month period versus the reference period (i.e., 13-24 months before the index bariatric surgery), as calculated with the negative binomial regression model with generalized estimating equations.

†Number of acute care use (ED visit or unplanned hospitalization) for HTN-related disease.
