# Supplementary material for: Association of bariatric surgery with risk of acute care use for hypertension-related disease in obese adults: population-based self-controlled case series study
Source: BMC Med. 2017 Aug 23;15:161. doi: 10.1186/s12916-017-0914-5 (PMC5568280; doi:10.1186/s12916-017-0914-5)
Supplement: Supplementary file 4 — Number of patients and risk of acute care use for hypertension-related disease, with more restrictive definition. (DOCX 27 kb) [file 12916_2017_914_MOESM4_ESM.docx]

**Additional file 4. Number of Patients and Risk of Acute Care Use for Hypertension-related Disease, with More Restrictive Definition**

|  | **Number of patients** | **Risk, % (95% CI)*** | **aOR (95% CI)**† | **P value** |
| --- | --- | --- | --- | --- |
| **Time interval** | (n=847) |  |  |  |
| 13-24 months before bariatric surgery | 141 | 16.6 (14.1-19.2) | reference | - |
| 1-12 months before bariatric surgery | 158 | 18.7 (16.0-21.3) | 1.13 (0.89-1.42) | 0.32 |
| 0-12 months after bariatric surgery | 91 | 10.7 (8.7-12.8) | 0.64 (0.49-0.83) | 0.001 |
| 13-24 months after bariatric surgery | 106 | 12.5 (10.3-14.7) | 0.74 (0.58-0.96) | 0.02 |

CI, confidence interval; aOR, adjusted odds ratio

*At least one acute care use (ED visit or unplanned hospitalization) for HTN-related disease as defined by the *ICD-9-CM* diagnosis codes 401 and 437.2.

†Adjusted odds ratios are for each 12-month period versus the reference period (i.e., 13-24 months before the index bariatric surgery), as calculated with conditional logistic regression.
