# Supplementary material for: Association of bariatric surgery with risk of acute care use for hypertension-related disease in obese adults: population-based self-controlled case series study
Source: BMC Med. 2017 Aug 23;15:161. doi: 10.1186/s12916-017-0914-5 (PMC5568280; doi:10.1186/s12916-017-0914-5)
Supplement: Supplementary file 5 — Number of patients and risk of acute care use for hypertension-related disease with gastric bypass and gastric banding. (DOCX 27 kb) [file 12916_2017_914_MOESM5_ESM.docx]

**Additional file 5. Number of Patients and Risk of Acute Care Use for Hypertension-related Disease with Gastric Bypass and Gastric Banding**

| **Time interval and type of bariatric surgery** | **Number of patients** | **Risk, % (95% CI)*** | **aOR (95% CI)**† | **P value** |
| --- | --- | --- | --- | --- |
| **Gastric bypass** | (n=655) |  |  |  |
| 13-24 months before surgery | 118 | 18.0 (15.1-21.0) | reference | - |
| 1-12 months before surgery | 119 | 18.2 (15.2-21.1) | 1.01 (0.78-1.31) | 0.95 |
| 0-12 months after surgery | 65 | 9.9 (7.6-12.2) | 0.54 (0.40-0.73) | <0.0001 |
| 13-24 months after surgery | 84 | 12.8 (10.3-15.4) | 0.70 (0.53-0.94) | 0.02 |
|  |  |  |  |  |
| **Gastric banding** | (n=270) |  |  |  |
| 13-24 months before surgery | 47 | 17.4 (12.9-22.0) | reference | - |
| 1-12 months before surgery | 50 | 18.5 (13.9-23.2) | 1.07 (0.71-1.61) | 0.75 |
| 0-12 months after surgery | 32 | 11.9 (8.0-15.7) | 0.67 (0.42-1.06) | 0.08 |
| 13-24 months after surgery | 33 | 12.2 (8.3-16.2) | 0.69 (0.43-1.09) | 0.11 |

CI, confidence interval; aOR, adjusted odds ratio

*At least one acute care use (ED visit or unplanned hospitalization) for HTN-related disease.

†Adjusted odds ratios are for each 12-month period versus the reference period (i.e., 13-24 months before the index bariatric surgery), as calculated with conditional logistic regression.
